# Characterization of *ex vivo* cultured neuronal- and glial- like cells from human idiopathic epiretinal membranes

**DOI:** 10.1186/1471-2415-14-165

**Published:** 2014-12-23

**Authors:** Sofija Andjelić, Xhevat Lumi, Xiaohe Yan, Jochen Graw, Morten C Moe, Andrea Facsk ó, Marko Hawlina, Goran Petrovski

**Affiliations:** Eye Hospital, University Medical Centre, Grablovičeva ulica 46, 1000 Ljubljana, Slovenia; Helmholtz Center Munich - German Research Center for Environmental Health, Institute of Developmental Genetics, Ingolstaedter Landstr. 1, D-85764 Neuherberg, Germany; Center for Eye Research, Department of Ophthalmology, Oslo University Hospital and University of Oslo, and Norwegian Center for Stem Cell Research, Kirkeveien 166, 0407 Oslo, Norway; Department of Ophthalmology, Faculty of Medicine, University of Szeged, Korányi fasor 10-11, 6720 Szeged, Hungary; Stem Cells and Eye Research Laboratory, Department of Biochemistry and Molecular Biology and Apoptosis and Genomics Research Group of the Hungarian Academy of Sciences, Faculty of Medicine, University of Debrecen, Egyetem tér 1, 4032 Debrecen, Hungary

**Keywords:** Idiopathic epiretinal membrane, Glial- and neuronal cell growth, Pluripotency, Calcium dynamics, Acetylcholine, Mechanostimulation, GFAP, Nestin-1, Sox2, Pax2

## Abstract

**Background:**

Characterization of the neuro-glial profile of cells growing out of human idiopathic epiretinal membranes (iERMs) and testing their proliferative and pluripotent properties *ex vivo* is needed to better understand the pathogenesis of their formation.

**Methods:**

iERMs obtained during uneventful vitrectomies were cultivated *ex vivo* under adherent conditions and assessed by standard morphological and immunocytochemical methods. The intracellular calcium dynamics of the outgrowing cells was assessed by fluorescent dye Fura-2 in response to acetylcholine (ACh)- or mechano- stimulation.

**Results:**

The cells from the iERMs formed sphere-like structures when cultured *ex vivo*. The diameter of the spheres increased by 5% at day 6 and kept an increasing tendency over a month time. The outgrowing cells from the iERM spheres had mainly glial- and some neuronal- like morphology. ACh- or mechano- stimulation of these cells induced intracellular calcium propagation in both cell types; in the neuronal-like cells resembling action potential from the soma to the dendrites. Immunocytochemistry confirmed presence of glial- and neuronal cell phenotype (GFAP and Nestin-1 positivity, respectively) in the iERMs, as well as presence of pluripotency marker (Sox2).

**Conclusion:**

iERMs contain cells of neuronal- and glial- like origin which have proliferative and pluripotent potential, show functionality reflected through calcium dynamics upon ACh and mechano- stimulation, and a corresponding molecular phenotype.

## Background

Epiretinal membranes (ERMs) are collections of cells and extracellular matrix that occur on the vitreoretinal interface of the central retina and are laid upon the inner limiting membrane (ILM). They have contractile properties and can lead to visual disturbance and metamorphopsia (distorted vision) due to their tractional effect on the underlying retina. The membranes comprise of glial cells, retinal pigment epithelial (RPE) cells, macrophages, fibrocytes and collagen found in varying proportions in accordance with their etiology. Idiopathic epiretinal membranes (iERMs) occur without any association with disease or disease history and are among the most common types of ERMs [1]. Cells of glial origin predominate in the iERMs [2].

It is thought that the iERMs form initially as a result of cell migration (RPE and glial cells such as Müller cells and astrocytes) from within the retina. These cells can either migrate through a hole or tear in the retina onto its surface, as is generally assumed to be the case for RPE cells, or they can simply extend processes out of the retina, as is the case for glial cells [1, 2]. Once the scaffold for their growth has been formed or the existing ILM has been populated by the migrating cells, other cell types present at the vitreoretinal interface, such as hyalocytes - the cells of the vitreous body, and macrophages may come into the picture [3]. Cell contact and attachment to the retina can cause proliferation and formation of sheets of membranes over its surface [2].

Little is known about the presence of neuronal-like cells and their function in human iERMs. Determining the exact cell types found in the membranes has been the focus of several previous studies [4–9]. Using light or electron microscopy, it has been shown that many cells seem to change their morphological characteristics as the ERMs develop, making it difficult to identify their origin [5–8]. In earlier studies [5, 10, 11], such cells were identified as myofibroblasts, hyalocytes, fibrous astrocytes, RPE, and macrophages using only morphology as the only means for identification. With the addition of immunocytochemistry, however, it has become apparent that the most abundant cell types found in the membranes are glia, macrophages, RPE cells, and fibroblasts [7, 9, 12, 13].

Previously, no reports of *ex vivo* culturing of cells coming out of iERMs and their functional and molecular characterization have been described. Most of the published studies present a microscopic observation of cells found in the structure of ERMs. We postulate that transdifferentiation of cells involved in the process of ERM formation might be more frequent. We have taken iERM material confirmed by clinical examination and optical coherence tomography (OCT), and cultivated it under adherent conditions. The proliferation potential and the size of the outgrowing cells or spheres were followed over a period of time. Although neuronal-like cells have not yet been detected directly in ERMs, some evidence exists for presence of Nestin-1 positive neural progenitor-like cells in these membranes [14]. Those studies emphasize the capacity of Müller cells, the predominant retinal glial cells, to express Nestin-1 in response to different acute damage such as experimental retinal detachment [15], and hypothesize that Müller cells are able to re-differentiate into retinal neurons after a neurodegenerative disease [16]. Furthermore, the functionality of the iERM outgrowing cells was hereby studied by assessing intracellular calcium [Ca^2+^]_i_ dynamics upon acetylcholine (ACh)- or mechano- stimulation; such changes play an important role in the regulation of cell function and affect every aspect of the cells’ life and death [17].

## Methods

### Ethics statement

All tissue collection complied with the guidelines of the Helsinki Declaration and was approved by the National Medical Ethics Committee of Slovenia. Patients’ anonymity and informed consent were provided in each case.

### Patient selection

A case-series study was performed on ten (10) patients in which vitrectomy was carried out due to presence of iERM as confirmed by OCT. The indication for surgery was a decline of visual acuity (VA <0.5 on Snellen charts) or better VA in cases when symptomatic metamorphopsia affected the daily activities. Surgeries were performed between May, 2012 and April, 2013 at the Eye Hospital, University Medical Centre Ljubljana, Slovenia. Only patients with iERM were included, and those with secondary or non-idiopathic ERM, as well as diabetic retinopathy, glaucoma, corneal scars, patients after rhegmatogenous retinal detachment, cataract and any other ocular conditions affecting the VA were excluded. Assessment of the VA, slit lamp biomicroscopy and fundus examination by indirect ophthalmoscopy was performed in each patient pre-operatively, and up to 6 month post-operatively. The patient history included age, sex, side of the eye to be operated (right or left eye). Using the Lens Opacities Classification System III classification, patients classified as having nuclear opacities were excluded, if the nuclear opalescence was grade  ≥2.5.

Time-domain OCT imaging (3D OCT 1000 Topcon, Japan) was performed in all cases and at each visit. Central Foveal Thickness (CFT) and Total Macular Volume (TMV) were recorded pre-operatively on the surgery day, on the first post-operative day and 3 months post-operatively.

Patients were examined post-operatively at day 1, 2 and 3, 1 week, 1 month and 6 months after the surgery.The best corrected VA and intraocular pressure were measured at each visit.

### Tissue collection and growth analysis

iERMs were removed en-block using endgripping forceps. Immediately after removing, the excised tissue was placed into sterile microtubes filled with (Balanced Salt Solution +) BSS+. At the end of surgery, a 360^o^ inspection of the peripheral retina was performed by scleral depression and the vitreous cavity filled with BSS+. In the case of any peripheral retinal break, an endolaser retinopexy and air tamponade were performed. One part of the iERMs was cultivated e*x vivo* under adherent conditions in high glucose medium (DMEM , Sigma, No. 5671) supplemented with 10% fetal bovine serum (FBS). The size and the shape of the outgrowing cells (area of outgrowth) from the sphere-shaped iERMs were recorded and followed on a daily basis up to 8 days and throughout their continued growth for more than 3 months after the isolation.

### Immunohistochemical analysis of the iERMs

The remaining part from each isolated iERM underwent immunohistochemical analysis. In brief, the surgically removed iERMs were placed immediately in 4% paraformaldehyde for at least 24 hours. Then iERMs were washed in PBS/0.1% TritonX-100 (PBST) two times, and penetrated in 0.1 M Glycin/PBS. After washing in PBST, iERMs were incubated in primary antibody (rabbit GFAP, 1:500, Cat.No.9269 (Sigma-Aldrich, Germany); mouse Nestin, 1:500, Cat.No.561230 (BD Pharmingen, Germany); rabbit Pax2, 1:200, Cat. No.2549-1 (Epitomics, Germany); goat Sox2, 1:500, Cat.No.sc-17320 (Santa Cruz, Germany); rabbit Ki-67, 1:1000, Cat.No.6013874 (Novocastra, Germany); mouse CRALBP, 1:500, Cat.No.sc-376082 (Saanta Cruz, Germany)) or blocking solution (Control) for 24 hours at 4°C, followed by washing and incubation with secondary antibodies (rabbit Alexa Fluor ® 488, 1:250, Cat.No.A21206 (Invitrogen, Germany); goat Cy3, 1:250, Cat.No. 705-165-147 (Dianova, Germany); mouse Cy5, 1:250, Cat.No.715-175-150 (Jackson Immuno, Germany)) for 1 hour at room temperature. The samples were then incubated in DAPI for 20 minutes and placed on the slides with mounting medium. All the immunofluorescence pictures (single plane images and Z-stacks) were taken by Olympus confocal microscopy (Olympus, Hamburg, Germany) and analyzed by a FluoView software (Olympus). Below is a list of all antibodies used and their dilution.

### Calcium dynamics measurement in iERM outgrowing cells

At day 8, the cultures were loaded with acetoxymethyl (AM) ester of Fura-2 (Fura-2 AM; Invitrogen – Molecular Probes, Carlsbad, CA, USA), a free cytosolic calcium (Ca^2+^) sensitive dye which was dissolved in DMSO and suspended in 1.5 mL of culture medium (final working concentration: 8 µM). The Fura-2 AM loading was carried out at 37 oC, 5% CO_2_ for 40 min. After loading, the cultures were washed twice for 7 min with 3 mL of the physiological saline with (in mM): NaCl (131.8), KCl (5), MgCl2 (2), NaH_2_PO_4_ (0.5), NaHCO_3_ (2), CaCl_2_ (1.8), HEPES (10), glucose (10)), pH 7.24. The Petri dish was then mounted onto inverted microscope, Zeiss Axiovert S 100 (Carl Zeiss, AG, Oberkochen, Germany). In order to evoke responses from the cells, an agonist - acetylcholine (ACh; Sigma, USA) was applied in 25 µM concentration and diluted in physiological saline. The global application of ACh as well as its washout from the bath was driven simply by the hydrostatic pressure of a 35 cm water column and controlled manually by a Luer-lock stopcock (WPI) and applied through a polyethylene plastic tubing (inner diameter 2 mm) attached to the coarse micromanipulator. The excess bathing solution was removed by a suction line. To additionally test a response to mechanical stimuli, such stimulation was carried out with a tip of a glass micropipette mounted on a MP-285 micromanipulator (Sutter, Novato, CA, USA) was used. Image acquisition was done with the 12-bit cooled CCD camera SensiCam (PCO Imaging AG, Kelheim, Germany). The software used for the acquisition was WinFluor (written by J. Dempster, University of Strathclyde, Glasgow, UK). Microscope objectives used were: 10x/ 0.30 Plan-NeoFluar and 63x ⁄ 1.25 oil Plan-NeoFluar (Zeiss). The light source used was XBO-75 W (Zeiss) Xe arc lamp. The excitation filters used, mounted on a Lambda LS-10 filter wheel (Sutter Instruments Co.) were 360 and 380 nm (Chroma). Excitation with the 360 nm filter (close to the Fura-2 isosbestic point) allowed observation of the cells’ morphology and of the changes in the concentration of the dye, irrespective of changes in free cytosolic Ca^2+^ concentrations ([Ca^2+^]_i_), while the 360⁄380 nm ratio allowed visualization of the [Ca^2+^]i changes in the cytoplasm. Image acquisition, timing and filterwheel operation were all controlled by WinFluor software via a PCI6229 interface card (National Instruments, Austin, TX, USA). Individual image frames were acquired every 500 ms resulting in frame cycles being 1 second long (two wavelengths).

### Statistical analyses

The statistical analysis used was paired Student’s t-test (two tailed), which was conducted using SPSS software.

## Results

### Pre-operative and post-operative patient data

The iERMs were diagnosed clinically and by OCT in 10 patients (6 males and 4 females; age: 63 to 78 years (average age: 72.2 ± 4.2 years)). Four eyes were phakic and 6 were pseudophakic. Vitrectomy was performed in the right eye in 5 and the left eye in 5 patients.

All patients had reduced VA (0.02 to 0.6 on Snellen charts (mean VA: 0.31 ± 0.22)) and metamorphopsia pre-operatively. In all cases, OCT clearly detected the ERMs without any vitreoretinal traction. CFT was increased in each of the patients and ranged from 371 µm to 649 µm (mean thickness: 502.7 ± 81.15 µm). TMV ranged from 8.31 to 11.39 mm^3^ (mean value: 9.99 ± 1.10 mm^3^). The surgery was in all cases 23-gauge sutureless vitrectomy. There were no intra-operative or post-operative complications. In some of the patients, immediately after the ERM removal, mild petechial haemorrhages occurred on the retinal surface, which usually were not seen on the first post-operative day. In one patient, at the end of the surgery a peripheral retinal break was noticed for which laser retinopexy and air tamponade were performed. During the 6 months follow-up period the retina remained stable in the later case.

Post-operatively the vision improved in 9 eyes, the VA ranging from 0.1 to 0.7 (mean VA: 0.43 ± 0.23). One eye (patient) had worse vision after surgery: the VA was reduced from 0.6 pre-operatively to 0.5 post-operatively. The primary reason for the subsequent decrease in VA was the occurrence or progression of nuclear sclerosis of the crystalline lens. Post-operatively, the CFT was also reduced in all patients and ranged from 273 to 432 µm (mean: 336.2 ± 46.7 µm). The TMV decreased after surgery and ranged from 7.64 to 8.77 mm^3^ (average value: 8.16 ± 3.46 mm^3^). These pre-operative and post-operative differences were statistically significant (p < 0.05).

### Growth potential of the iERM outgrowing cells

**Figure 1:**
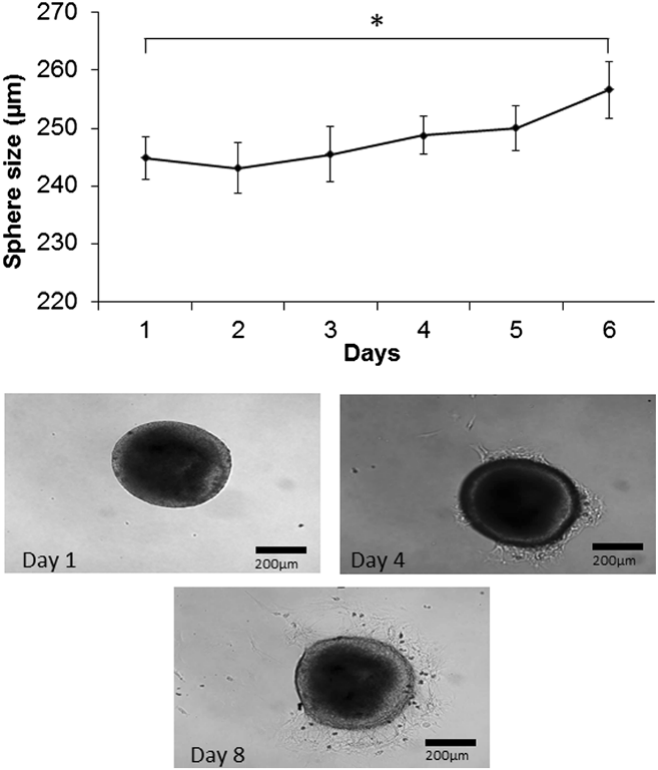
**Sphere-like growth of the cultivated iERMs.** Data shown are average of four independent experiments (*p = 0.03).

The cells from the iERMs formed sphere-like structures when cultured *ex vivo*. The diameter of the spheres increased significantly by 5% at day 6 (256.6 ± 90.0 µm, p = 0.03) and kept an increasing tendency over a month period. The cells growing out of the spheres had mainly a glial or star-shaped morphology with many processes coming out of the soma, while some had a neuronal- like morphology with dendritic- and axonal- like extensions coming out of the soma (Figure 1).

### Immunophenotype of the cells found in the iERMs

**Figure 2:**
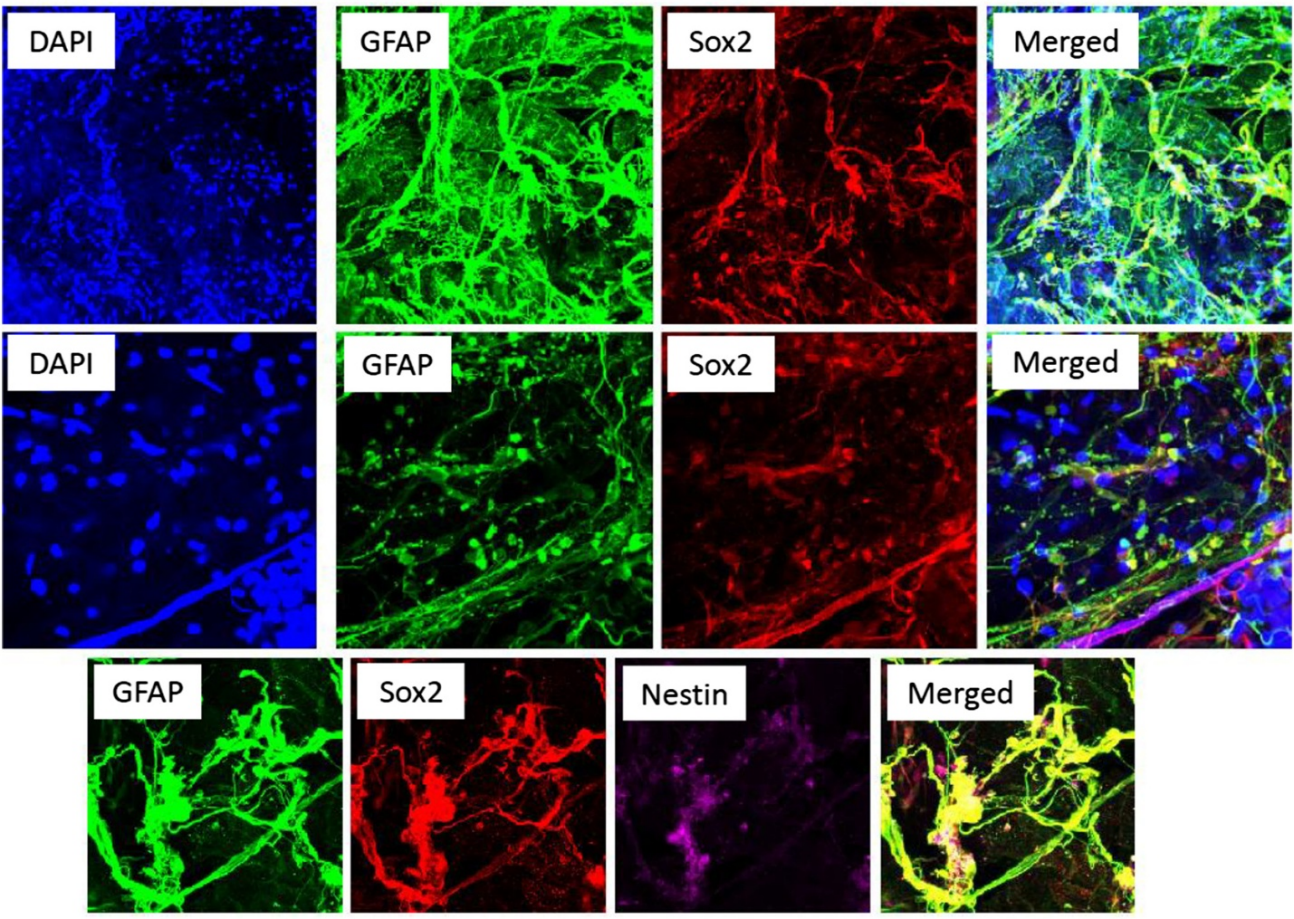
**Immunofluorescent images of iERMs stained for GFAP, Sox2 and Nestin-1.**

**Figure 3:**
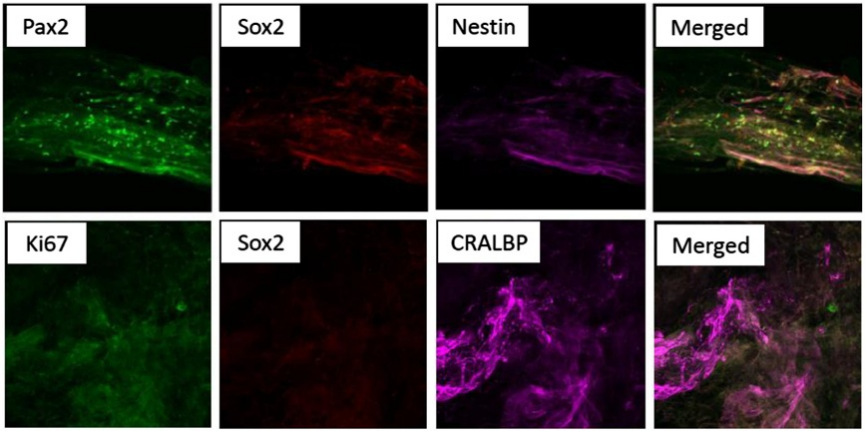
**Immunofluorescent images of iERMs stained for Pax2, Sox2 and Nestin-1.** (Cellular retinaldehyde-binding protein (CRALBP) serves as a marker for Müller glial cells of the retina).

The iERMs obtained from surgery were examined for expression of glial-, neuronal- and pluripotency markers. Most of the cells in the explanted iERMs expressed GFAP, while some expressed Sox2 and Nestin-1 (Figure 2). Few of the cells co-expressed GFAP and Sox (Figure 2, middle panel images) and similarly, few of them co-expressed GFAP, Sox2 and Nestin-1 at the same time (Figure 2, lower panel images).Only a trace amount of the cells expressed Ki-67 (Figure 3). Pax 2 (a developmental marker), was also detected in the iERMs, which was co-expressed with some of the Sox2 and CRALBP (Müller glial marker) positive cells, respectively (Figure 3).

### Functionality of the iERM outgrowing cells

**Figure 4:**
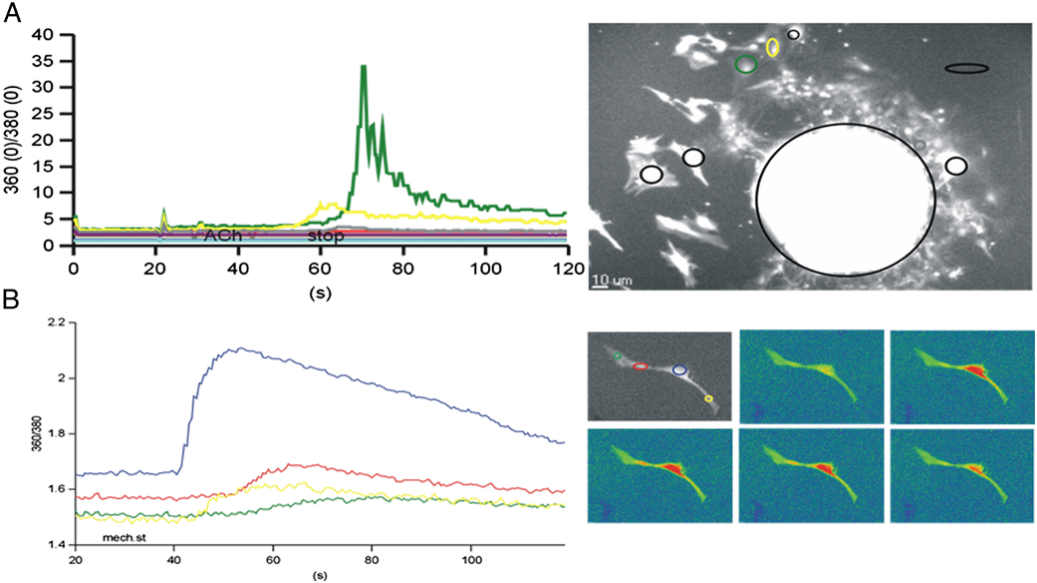
**Calcium dynamics in the neuronal-like cells derived from an iERM upon different stimulation.** [Ca^2+^]_i,_ responses upon acetylcholine (ACh) **(A)** and mechano- stimulation **(B)**.

The functionality of the iERM outgrowing cells was assessed by the [Ca^2+^]_i_ dynamics upon ACh or mechanical stimulation. Some cells derived from the iERM spheres responded to the pharmacological stimulation by ACh with an increase in [Ca^2+^]_i_, while others showed no change in the [Ca^2+^]_i_. The rise in [Ca^2+^]_i_ was monophasic for the activated cells (Figure 4A,B). The outgrowing cells from the iERM spheres had mainly a glial- and some neuronal- like morphology. Stimulation of these cells with ACh induced [Ca^2+^]_i_ changes in the neuronal-like cells, while not in the glial-like cells (Figure 4A). Upon mechanical stimulation of the single neuronal-like cells with glass micropipette, [Ca^2+^]_i_ propagation could be observed from the cell body to the dendrites, resembling action potential (Figure 4B).

The cell morphology, as assessed by observation of 360 nm fluorescencent images, is shown on Figure 4A for the group of cells derived from the sphere of one patient, and for the single cells derived from the sphere of another patient (Figure 4B). The regions of interest (ROIs) are superimposed on the images of morphology, and are presented in different colors which correspond to the traces showing the time courses of the 360⁄380 ratio, proportional to [Ca^2+^]_i_ for the selected ROIs. The resting levels, the increase in 360/380 ratio upon stimulation and the amplitudes of the ratio of the responses, corresponding to the resting levels and the changes in [Ca^2+^]_i_ can be seen in Figure 4.

## Discussion

A case-series study of 10 patients with iERM is presented in which immunohistochemical and functional evidence of their *ex vivo* proliferative and pluripotency potential is shown. Apart from that, clinical data showing similar postoperative outcomes of VA and OCT changes to other previously published data are presented in support of the successful procedures carried [18–20].

To date, the pathogenesis of ERMs is not fully clear. The two main cellular components of the ERMs are cells of retinal and extra-retinal origin, as well as extracellular matrix (consisting of collagen, laminin, tenascin, fibronectin, vitronectin and thrombospondin) [9, 21]. The origin of the cells which contribute to ERM formation has not been fully understood. Hiscot et al. identified oval cells, spindle-shaped cells, and cuboidal cells in ERMs, which stained positively for epithelial markers [4]. Further histological and immunohistochemical analysis has shown that cells involved in ERM formation are RPE metaplastic cells, glial cells (Müller cells and astrocytes), hyalocytes, endothelial cells, fibroblasts, myofibroblasts, monocytes, and macrophages [7, 22]. Zhao et al. stated that hyalocytes constitute a major cell type of the epiretinal cell proliferation in eyes with macular pucker and vitreomacular traction syndrome. In the membranes, they noticed myofibroblast-like cells, being immunoreactive for α-SMA and of unknown origin, representing contractile elements of epiretinal tissue possibly as a consequence of cellular transdifferentiation [23].

The only cellular components of the ERMs in a series studied by Snead et al. were GFAP^+^ and AE1/AE3^+^ cells. They preferred to name these cells laminocytes as a term for glial cells, glial fibroblasts, astrocytic cells, and Muller cells [24]. Lewis et al. found the proof of neurite growth in ERMs since 100% of the samples contained neurofilament labelled processes [25].

Electron microscopy and immunocytochemical studies have shown changes of the morphological characteristics of cells during ERM development, but it was difficult to distinguish the origin of these cells from morphological studies only [6–8]. The outgrowth of Müller cell processes onto the inner retinal surface has been considered being the first step in the process of ERM formation [10, 26]. Schubert et al. suggested that under normal conditions many vitreous fibers distribute tractional forces to numerous Müller cell attachments. However, in cases of partial posterior vitreous detachment (PVD), fewer vitreous fibers and Müller cells endure most of the traction, which may result in a chronic irritation of Müller cells. PVD and chronic irritation of Müller cells can facilitate a local release of factors that, in turn, can induce Müller cell gliosis and vascular leakage [27]. General signs of Müller cell gliosis are cellular hypertrophy and upregulation of the intermediate filaments vimentin and GFAP, followed by transient or long lasting proliferation of these cells [12]. GFAP expression in simple ERMs demonstrates that the majority of the cells involved in the membrane formation derive from glial cells [13]. Within the membranes, glial and pigment epithelial cells may transdifferentiate to other cells, e.g. contractile myofibrocytes, which is characterized by a reduction in cell-specific proteins such as GFAP and cytokeratins [28, 29].

In their immunocytochemistry model of ERMs, Oberstein et al. showed the presence of Ki-67^+^ proliferating glial-, ezrin^+^ RPE-, GFAP^+^ and vimentin^+^ glial cells, as well as ricin^+^ immune cells in all four types of the ERMs studied, regardless of their duration of occurrence in the eye. According to their findings, there is strong evidence that RPE cells can migrate through the intact retina [3]. These data support the hypothesis that ERM formation is a wound healing process [30].

Bringmann et al. described how Müller cells in proliferative vitreoretinopathy (PVR) can display a distinct potential for transdifferentiation into a progenitor- or neuron-like phenotype [31]. They hypothesized that cellular transdifferentiation may represent one reason for the relatively few glial and pigment epithelial cells being detected in fibrocellular membranes by commonly used immunocytochemical markers. Progenitor cells have been found in tissues which have potential to regenerate and differentiate into various phenotypes. One marker for progenitor cells is Nestin. Mayer et al. found in adult human retina and ERMs removed during retinal surgery Nestin-staining patterns associated with cells of neural and glial morphology. Nestin positive cells in the adult human retina display morphological and geographical similarity to neurons, including retinal ganglion cells, other neurons, and Müller cells. In the ERMs, these cells were found co-expressed with GFAP [14].

In distinction to the histological and immunocytochemical studies published so far, we established a different model for analyzing the proliferative, differentiation, pluripotency and functional potential of the cells isolated from iERMs. The collected iERMs were cultivated *ex vivo* under adherent conditions in high glucose medium supplemented with 10% FBS. Cells from the ERM started to form sphere like structures within first few days and kept growing over a month period (the overall follow-up time was 6 months when the ongoing growth was intentionally discontinued). Within the first 6 days, the diameter of the growing spheres increased significantly, the cells showing mainly a glial or star-shaped morphology, while some showing neuronal-like morphology, which was clearly reflected by most cells expressing GFAP, and some expressing Nestin-1. In addition, few cells expressed Sox2, while some showed co-expression of Sox2 and Nestin-1. Only a trace amount of the cells expressed the cell proliferation marker Ki-67. Interestingly, Pax2 which is prominently expressed at sites where tissue outgrowth and shaping takes place [32] was expressed in the cells found in iERMs and it was found partially co-expressed with Sox2 positive cells.

In animal models, it has been shown that small increases in the levels of Sox2 expression (2-fold or less) could lead to rapid differentiation of epithelial stem cells into cells expressing neuroectodermal, mesodermal and trophectodermal markers [33]. Sox2 is probably one of the proteins which act as molecular rheostat in the control of epithelial stem cell self-renewal and pluripotency [34]. Demonstrating the presence of Sox2 in the iERMs proved the presence of pluripotency of at least some of the cells growing out of the iERMs. These data are unique in studying the proliferation of iERM cells altogether.

The neuronal-like cells in ERMs have not been found directly, so neither has been their calcium signaling. Nestin being an intermediate filament marker for neural progenitor cells has been shown to exist in ERMs [14], however, other studies have emphasized the capacity of Müller cells, the predominant retinal glial cells, to express Nestin in response to different acute damage paradigms such as experimental retinal detachment [15]. These studies have hypothesized that Müller cells are capable of re-differentiating into retinal neurons after a neurodegenerative disease [16]. The calcium signaling in outgrowing cells can be studied by chemical stimulation following administration of ACh, and mechanical stimulation, both being known to induce calcium signaling in retinal cells [35]. ACh receptors are widely present in the retina and they play important roles in it. Nicotinic ACh receptors, alpha7 nAChR, are expressed in RPE cells [36] and in bipolar, amacrine, and ganglion cells of the rabbit retina [37]. Muscarinic receptors, M1 and M4 mAChR, are expressed in retinal ganglion cells [38], and all five mAChR subtypes are expressed by subpopulations of bipolar, amacrine, and ganglion cells in the rabbit retina [39]. Amacrine cells, which are excitatory cells with an ACh release early in the development of the retina, have been show to require this neurotransmitter for development of retinal waves [40]. The transient cholinergic network plays an important role in the various aspects of retinal development [41]. Control of cell proliferation by neurotransmitters in the developing vertebrate retina also involves ACh [42]. Neuroprotection of rat retinal ganglion cells against glutamate-induced excitotoxicity is mediated through alpha7 nACh receptors [43].

The presence of ACh receptors on iERM cells and the calcium response of these cells to ACh stimulation, to the best of our knowledge, have not been studied before. As iERMs appear to form initially as a result of cells coming from within the retina, and since ACh plays an important role in retinal development, altogether, this suggests a putative role of ACh in the iERM development, which we studied further. Nicotinic ACh receptors have multiple roles in proliferation in cancer in general [44], suggesting they might have a similar role in the proliferation of iERM cells. Recent studies have documented the effect of topical nicotine on improving dermal wounds [45] suggesting it may have roles in wound repair, therefore, possibly cell repair as well. Blocking n- and m-AChRs on non-innervated cells causes cellular dysfunction and/or cell death. Embryonic stem cells from mice, epithelial, endothelial and immune cells can all synthesize ACh, which via differently expressed patterns of n- and mAChRs can modulate cell activities to respond to internal or external stimuli. This helps maintain and optimize cell function, such as proliferation, differentiation, formation of a physical barrier, migration, and ion and water movements [46].

Alternatively, when cells are stimulated by mechanical deformation, in particular, when diseases characterized by mechanical stress cause stretching of the RPE cells by the vitreous or hyperplastic membranes' formation as in rhegmatogenous retinal detachment and PVR, the ‘on’ mechanisms become activated and the cytosolic free Ca^2+^ concentration increases. The Ca^2+^ ‘on’ mechanisms can include channels located at the plasma membrane which can further regulate the inexhaustible supply of Ca^2+^ from the extracellular space, and channels on the endoplasmic and sarcoplasmic reticulum which release the finite intracellular Ca^2+^ stores [47]. Mechanical stimulation of iERM cells has not been described so far, although mechanical stimulation of retinal cells has shown changes in the [Ca^2+^]_i_ levels. Mechanical stimulation of astrocytes of rat retina could initiate an increase in the [Ca^2+^]_i_ that propagated through astrocytes and Müller cells as intercellular waves. Generation of Ca^2+^ waves can occur by the release of Ca^2+^ from internal stores, but the waves do not evoke changes in cell membrane potential. It was suggested that such glial Ca^2+^ waves may constitute an extraneuronal signaling pathway [48, 49]. Forces applied to resting primary astrocytes produced a fast transient change of [Ca^2+^_]i_ adapting within seconds [50]. Furthermore, stimulation of RPE cells with mechanical stress can upregulate matrix metalloproteinases (MMP-2) and fibronectin (FN) expression through activation of the p38 pathway [51].

Elevated intraocular pressure can also trigger a physiological release of ATP from the retina. Stimulation of the purinergic P2X_7_ receptor for ATP on retinal ganglion cells can lead to elevation of [Ca^2+^]_i_ and excitotoxic death [52]. Isolated retinal ganglion cells can respond directly to mechanical deformation with pannexin-mediated ATP release and autostimulation of P2X_7_ receptors [53].

Since calcium signaling can occur in both neuronal and glial cells, calcium imaging cannot be used for discriminating glia cells from neurons [54–56], which can be an obvious limitation of this study. Glial cells can respond to various stimuli with an increase in [Ca^2+^]_i_, but not by forming action potentials. Mechanical stimulation of astrocyte somata in rat retina could evoke calcium waves that could propagate through both astrocytes and Müller cells [49]. Calcium signaling upon ACh stimulation has been demonstrated in cultured retinal neurons and Müller cells [57]. Forces applied to resting primary astrocytes produce a fast transient change of [Ca^2+^]_i_ adapting within seconds [50]. ACh receptors are known to exist in astrocytes of rat hippocampus and cortex [58, 59] and to induce the increase in [Ca^2+^]_i._

Overall, the etiology, cellular types and the origin of the cells found in ERMs has been a matter of long-term debate. Investigations searching for cell type–specific markers expressed on ERM cells, in particular coming from iERMs, have not lead to a distinct conclusion about their origin, probably due to phenotypic transdifferentiation and lack of a specific marker for transdifferentiated cells thereof [7]. A majority of the present cells in this study were GFAP^+^, however some Sox2^+^ and Ki-67^+^ cells, as well as Pax2^+^ (a putative new marker for cells found in iERMs) could be detected in the studied membranes. Therefore, we postulate that cells found in iERMs have a proliferative and differentiation or transdifferentiation potential [23] based upon their immunocytochemical properties and growth patterns, and the functional studies with chemo- and mechano- stimulation. Further functional studies as well as protein and gene expression studies are needed to find out which ACh receptor types in iERMs are affected by application of different agonists and antagonists, and which one of them can further affect the proliferation and differentiation potential of the ERM cells.

## Conclusions

Outgrowing cells from human iERMs show mainly glial- and some neuronal- like phenotype (GFAP and Nestin-1 positivity, respectively), proliferate and express pluripotency potential (Sox2 positivity). ACh- or mechano- stimulation of these cells induces intracellular calcium propagation; in the neuronal-like cells, it resembles action potential from the soma to the dendrites.

## Abbreviations

ACh: Acetylcholine
[Ca^2+^]: AM: Acetoxymethyl ester; intracellular calcium
CFT: Central Foveal Thickness
CRALBP: Cellular retinaldehyde-binding protein
FN: Fibronectin
FBS: Fetal bovine serum
GFAP: Glial fibrillary aacidic protein
iERM: Idopathic epiretinal membranes
MMP-2: Matrix metalloproteinase 2
OCT: Optical coherence tomography
PBST: PBS/0.1%TritonX-100
PVD: Posterior vitreous detachment
PVR: Proliferative vitreoretinopathy
ROI: Region of interest
RPE: Retinal pigment epithelium
TMV: Total Macular Volume
VA: Visual Acuity.
